# New developments in women’s health research and implications for nursing

**DOI:** 10.4069/whn.2025.12.16

**Published:** 2025-12-31

**Authors:** Sue Kim

**Affiliations:** Mo-Im Kim Nursing Research Institute, College of Nursing, Yonsei University, Seoul, Korea

This editorial reflects on 30 years of publishing *Women’s Health Nursing* (WHN) as the official journal of the Korean Society of Women Health Nursing, while sharing new developments in women’s health research that has implications for nursing. I also share journal metrics released in 2025.

## WHN’s 30^th^ year milestone

In celebration of 30 years of the journal, we successfully published a special issue on “Digital health interventions (DHI) for women’s health” in June 2025 (https://www.e-whn.org/current/index.php?vol=31&no=2). This was a timely follow-up of our 2023 special issue on “Digital era education for women’s health and wellbeing” (https://www.e-whn.org/current/index.php?vol=29&no=3). This year's special issue on DHI presented the state of gender in digital health and the importance of being gender intentional when considering digital determinants of health [1]. Highlighting the role of digital health literacy in bridging the gender gap in DHI [2] was another timely contribution. The special issue also included systematic reviews on DHI for high-risk pregnancies [3], managing menstrual symptoms [4], and DHI for oncofertility [5], as well as studies that used a mobile application for breastfeeding [6], labor nursing simulation using an artificial intelligence tutor [7], and analyzing pregnant women’s eHealth literacy and attitudes towards internet health information [8]. We hope readers will note the findings of these timely studies and apply them when developing and implementing DHI for women. Among our other publications covering diverse health issues for women this year, much interest was observed for a paper that applied Levine’s conservation model in examining how women with uterine fibroids intending to maintain fertility commonly face multidimensional and interrelated health challenges, especially for symptom severity, fatigue, and sexual function [9]. We look forward to more studies that actively use and expand theoretical frameworks.

## New developments for women’s health research

Among the notable developments in research this year, I wish to mention two that nurse researchers should be aware of, especially as we seek to impact women’s health nursing. The first is intersectional analysis for science and technology [10], which has direct applicability to health research. The concept of intersectionality “prompts researchers to consider the compounded disadvantages or advantages tied to sociopolitical dimensions, such as ethnicity, sex, gender, class, educational status or migration status, within specific social and environmental contexts” [10]. Intersectional factors are proposed at three interconnected levels—sociopolitical dimensions, contextual domains, and environmental conditions—which can enhance accuracy and efficiency in research and offer clearer insight compared to assumptions of independent influence and non-intersectional analyses that have been predominantly oservable in health science research [10]. Nurse researchers can start by familiarizing themselves with the recently published guidelines for intersectional analysis [11] and conduct thorough literature reviews to identify and select the relevant intersectional factors for the main outcome. Utilizing statistical methods that allow for and highlight intersectional analysis [10] is also recommendable.

The second development is the guideline updates for reporting protocols for randomized controlled trials (RCT), i.e., the 2025 SPIRIT guidelines [12]; and for reporting RCT findings, i.e., the 2025 CONSORT [13,14]. I hope nurse researchers keep abreast of these recent updates.

## Journal metrics

In terms of journal impact factors (JIF), the journal was indexed in emerging sources citation index (ESCI) in 2022 under its former title, *Korean Journal of Women Health Nursing*. The 2024 JIF (released in June 2025) was 1.2, an increase compared to the 2023 JIF of 1.0. Because the journal transitioned to its current title in 2024, we expect to receive JIF as WHN in the coming years. As for other parameters this year, our Scopus CiteScore is 1.8 and Scimago 2024 SJR (the first report since our new title of *Women’s Health Nursing*) is 0.26, suggesting rich opportunity for reaching a wider audience. We will continue to strive for excellence in scholarly publishing for wider impact in improving women’s health through nursing research.

This year’s basic statistics are presented in Table 1. A total of 65 manuscripts were submitted to WHN this year, of which 55 were unsolicited manuscripts from 14 countries (excluding South Korea). This is an increase from 2024 and with 19 out of the 55 (34.5%) submissons being from overseas, continuous ripple effects are noted since being indexed in Pubmed Central and ESCI in 2022. The acceptance rate for 2025 was 64.7%, a slight increase compared to 61% in 2024 [15].

I wish to emphasize that WHN, a reputable open-access journal indexed in all four major databases (MEDLINE, Scopus, PMC, ESCI), offers excellent editorial quality at reasonable fees and is demonstrating widening scholarly impact. We look forward to quality submissions that can contribute to the health of women.

## Appreciation for Editors and 2025 Reviewers

WHN is endebted to our tireless editors (marked with *) and the following dedicated reviewers who have supported the journal this year:

Bae, Kyungeui (Dongseo University)

Chae, Hyun Ju (Sangmyung University)

Chae, Hyunju (Sangmyung University)

Cho, Ok-Hee (Kongju National University)

Choi, Hyunkyung* (Kyungpook National University)

Choi, So Young (Gyeongsang National University)

Chung, Chae Weon* (Seoul National University)

Chung, Mi Young (SunMoon University)

Go, Gee Youn (Woosuk University)

Ha, Ju Young (Pusan National University)

Haruna, Megumi* (University of Tokyo, Japan)

Han, Jeehee (Chung-Ang University)

Hong, Sehoon (CHA University)

Hwang, Kyung Hye (Suwon Science College)

Hwang, Sung Woo (Baekseok Culture University)

Jeong, Geum Hee (Hallym University)

Kang, Hyunwook (Kangwon National University)

Kang, Minkyung (Ajou University)

Kang, Saem Yi (Gachon University)

Kang, Sook Jung* (Ewha Womans University)

Kim, Hae Won (Seoul national University)

Kim, Hye Young (Keimyung University)

Kim, Hyun Kyoung* (Kongju National University)

Kim, Ju Hee* (Kyung Hee University)

Kim, Kyung-Ah (Incheon Catholic University)

Kim, Kyung-Won (Daegu Haany University)

Kim, Mi Jong (Hannam University)

Kim, Moon-Jeong (Pukyong National University)

Kim, Myoung Hee (Semyung University)

Kim, Seonho (Chungbuk National University)

Kim, Su Hyun (Mokpo National University)

Kim, Sue* (Yonsei University)

Kim, Sun-Hee (Daegu Catholic University)

Kim, Yoonjung (Hallym University)

Kim, Youngju (Daejeon Health Institute of Technology)

Ko, Eun (Sunchon National University)

Kwon, Yoo Rim (Ansan University)

Lee, Jisan (Gangneung–Wonju National University)

Lee, Ju-Young* (The Catholic University)

Lee, Sun Hee (Baekseok Culture University)

Loke, Alice Yuen* (Hong Kong Polytechnic University, Hong Kong)

Nho, Ju-Hee* (Jeonbuk National University)

Park, Meera (Changshin University)

Park, Seo-A (Kyungwoon University)

Park, Sihyun* (Chung-Ang University)

Park, So Mi (Yonsei University Wonju)

Shin, Gi Soo* (Chung-Ang University)

Shorey, Shefaly* (National University of Singapore, Singapore)

Song, Ju-Eun* (Ajou University)

Stevenson, Eleanor* (Duke University, USA)

Yeom, Gye Jeong (JEI University)

Yoo, Hana (Daejeon University)

We sincerely congratulate the following “Reviewers of the Year 2025” for their dedication in promoting women’s health nursing scholarship through peer reviewing.

• Saemi Kang (Gachon University)

• Yoonjung Kim (Hallym University)

• Mi Young Chung (Sun Moon University)

And finally, special recognition goes to our “Editor’s Pick 2025 Award”, as the most cited manuscripts in the Scopus and Web of Science databases for the period of 2023-2024.

• Song YA, Kim M. Effects of a virtual reality simulation integrated with problem-based learning on nursing students' critical thinking ability, problem solving ability, and self-efficacy: a non-randomized trial. Korean J Women Health Nurs. 2023 Sep;29(3):229-238. English. doi: 10.4069/kjwhn.2023.09.12.

• Park CG. Implementing alternative estimation methods to test the construct validity of Likert-scale instruments. Korean J Women Health Nurs. 2023 Jun;29(2):85-90. doi: 10.4069/kjwhn.2023.06.14.2.

It has been a privilege to serve as editor-in-chief for the past six years, which was a period of immense professional and personal growth. I am endebted to past and current presidents of the Korean Society of Women Health Nursing and the many editors who have tirelessly shared their time and expertise. WHN looks forward to new reviewers from near and far, with whom we wish to step into and co-create the journal's next developments. Please contact us at whn@e-whn.org with questions, comments, and requests to join as reviewer.

## Figures and Tables

**Table 1.** Basic statistics on manuscripts submitted to *Women’s Health Nursing* in 2025

Category	Data	Notes
Commissioned manuscripts (n)	10	3 Editorials, 4 *Issues & Perspectives, *3 Invited papers
Unsolicited manuscripts (n)	55	
Accepted manuscripts (n)	28	
Non-accepted manuscripts (n)	19	5 Rejected, 11 rejected before review (unsuitable), 3 withdrawn
Manuscripts reviewed and determined (n)	33	28 Accepted, 5 rejected
Manuscripts under review or revision (n)	18	
Acceptance rate (%)	65	33/51=0.647
